# Expected total thyroxine (TT4) concentrations and outlier values in 531,765 cats in the United States (2014–2015)

**DOI:** 10.1371/journal.pone.0213259

**Published:** 2019-03-06

**Authors:** Maya Lottati, David Aucoin, David S. Bruyette

**Affiliations:** 1 VCA Veterinary Specialists of the Valley- Department of Internal Medicine, Woodland Hills, CA, United States of America; 2 VCA Antech, Los Angeles, CA, United States of America; 3 Anivive Lifesciences, Long Beach, CA, United States of America; Texas A&M University College of Veterinary Medicine, UNITED STATES

## Abstract

**Background:**

Levels exceeding the standard reference interval (RI) for total thyroxine (TT4) concentrations are diagnostic for hyperthyroidism, however some hyperthyroid cats have TT4 values within the RI. Determining outlier TT4 concentrations should aid practitioners in identification of hyperthyroidism. The objective of this study was to determine the expected distribution of TT4 concentration using a large population of cats (531,765) of unknown health status to identify unexpected TT4 concentrations (outlier), and determine whether this concentration changes with age.

**Methodology/Principle findings:**

This study is a population-based, retrospective study evaluating an electronic database of laboratory results to identify unique TT4 measurement between January 2014 and July 2015. An expected distribution of TT4 concentrations was determined using a large population of cats (531,765) of unknown health status, and this in turn was used to identify unexpected TT4 concentrations (outlier) and determine whether this concentration changes with age. All cats between the age of 1 and 9 years (n = 141,294) had the same expected distribution of TT4 concentration (0.5–3.5ug/dL), and cats with a TT4 value >3.5ug/dL were determined to be unexpected outliers. There was a steep and progressive rise in both the total number and percentage of statistical outliers in the feline population as a function of age. The greatest acceleration in the percentage of outliers occurred between the age of 7 and 14 years, which was up to 4.6 times the rate seen between the age of 3 and 7 years.

**Conclusions:**

TT4 concentrations >3.5ug/dL represent outliers from the expected distribution of TT4 concentration. Furthermore, age has a strong influence on the proportion of cats. These findings suggest that patients with TT4 concentrations >3.5ug/dL should be more closely evaluated for hyperthyroidism, particularly between the ages of 7 and 14 years. This finding may aid clinicians in earlier identification of hyperthyroidism in at-risk patients.

## Introduction

Since its first description in 1979 (Peterson M.E., Johnson J.G., Andrews L.K. Spontaneous hyperthyroidism in the cat. [abstract]. American College of Veterinary Internal Medicine. Seattle WA, 1979:108), the prevalence of hyperthyroidism has dramatically increased making it the most commonly diagnosed endocrinopathy in the cat [1–3]. The diagnosis of hyperthyroidism relies on evidence of thyroid hyperfunction (presence of compatible clinical signs, a palpable thyroid nodule, and elevated total thyroxine (TT4) concentrations) [4]. Clinicians rely heavily upon the TT4 concentration reference interval (RI) provided by diagnostic laboratories to make diagnostic and therapeutic decisions. These RIs are generally based on a relatively small population of healthy, young cats, a practice which is commonplace in veterinary medicine [5]. Reference intervals are generally determined by the nonparametric method of percentile estimates with confidence intervals to determine the central 95th percentile interval (i.e., 2.5^th^ through 97.5^th^ percentile range) for results from > 120 clinically normal subjects.

In studies where the health of the patients cannot be established, the data is often evaluated using Tukey’s method [6], which is appropriate for analyzing large univariate data sets. This methodology is valid and useful for establishing expected and unexpected values in a large population of patients, even when data is derived from patients with an unknown health status [7–8]. For each data set the median is defined as the middle value of the distribution where 50% of data results are higher and 50% are lower. The interquartile range (IQR) is defined as the range of values that reside in the middle 50% of concentrations between the lower quartile (25^th^ percentile) and upper quartile (75^th^ percentile). The expected distribution of TT4 concentration is defined as those contained within an upper value (1.5*IQR+ upper quartile) and a lower value (1.5*IQR-lower quartile). An unexpected TT4 concentration signifies an outlier, even if the value is within the laboratory’s RI, and is defined as a data point that is outside of the expected concentration for the sampled population [9–10].

Clinicians frequently encounter patients with compatible physical examination findings (enlarged thyroid) and/or clinical signs of hyperthyroidism but with TT4 concentrations within the laboratory’s RI. Such cats may be truly normal (non-hyperthyroid) or abnormal (hyperthyroid with concurrent non-thyroidal disease or early, emerging disease) requiring additional conformational testing or monitoring [11]. The identification of an unexpected TT4 value above which patients are considered statistical outliers would allow practitioners to recognize potentially hyperthyroid patients even when values come back within the RI. Recognizing unexpected TT4 concentrations may be a clinically useful piece of information, as it may prompt the clinician to pursue additional endocrine tests in a cat with clinical signs of hyperthyroidism or more frequently monitor an asymptomatic cat that may eventually progress to hyperthyroidism. Previous authors have suggested that values in the upper 33–50% of the RI in patients suspected as being hyperthyroid should undergo additional testing but the basis for these recommendations has not been validated in a large population and no specific cut-offs have been available [12–14].

The objectives of the study reported here were to 1) Determine the expected distribution of TT4 concentration and outlier values based on a large population of cats encompassing all ages and breeds in the US, 2) compare these TT4 concentrations to the standard laboratory RI, and 3) determine if and when the unexpected TT4 concentration changes with age.

## Materials and methods

### Study design

Retrospective, population-based review and analysis of laboratory results in adult cats for which a unique TT4 concentration was measured.

### Selection criteria

An electronic database of laboratory results from a large reference diagnostic laboratory (Antech Diagnostics Laboratory; Irvine, CA) was reviewed between January 2014 and July 2015 from both general practice and specialty veterinary hospitals in the US. Data provided was anonymized with each patient being assigned a unique identification number based on the patient’s name and veterinary clinic. Information obtained included TT4 result and patient signalment (age, sex, breed, and reproductive status). Client information was not included. If multiple TT4 concentrations were measured in a given patient in the same hospital during the study period, only the initial measurement was included to assist in excluding subsequent samples from ill patients with multiple laboratory submissions and hyperthyroid cats with multiple TT4 measurements following the institution of therapy. Patients between the age of 1 week and 1 year were categorized as < 1 year of age. TT4 concentrations from cats over the age of 20 years, TT4 concentrations greater than 20 ug/dL, and samples for which there was no assigned age or sex were excluded from the data set due to their low frequency and/or high likelihood of error. All age groups were included to assess the effect of age on both expected and unexpected TT4 concentrations and to document if and when the unexpected TT4 concentration change with age.

### Thyroxine (TT4) hormone assay

All serum TT4 concentrations were measured by the same homogenous thyroxine enzyme immunoassay (DRI Thyroxine Assay, Microgenics Corporation; Freemont, CA; EIA), which has been previously validated for measuring TT4 concentrations in cats [15–16]. Guidelines provided by the American Society for Veterinary Clinical Pathology, which are based on the 2008 Clinical Laboratory and Standard Institute recommendations for determination of RIs in a veterinary species, were followed in the present study. During the time samples were collected and analyzed for this study all Antech laboratories in the United States were measuring TT4 using the same EIA assay. This assay was validated in 2 different Antech laboratories (Irvine, CA^15^ and Long Island, NY)[16] for use in all Antech laboratories measuring TT4. In the Irvine laboratory, the following results were obtained: All serum TT4 concentrations were measured by the same homogenous thyroxine enzyme immunoassay (EIA). The lower limit of detection for this assay was 0.5 ug/dL, with an upper limit of 20 ug/dL. The assay was validated internally by the laboratory using serum from 20 cats with a wide range of TT4 values. Intra-assay coefficients of variation (CV) were 4.3% (at mean T4 concentration, 4.21 ug/dL) and 2.3% (at mean T4 concentration, 14.13 ug/dL). Interassay CV was 8% (at mean T4 concentration 3.73 ug/dL). The reference interval for this assay (0.8–4.0 ug/dl) was established by the nonparametric method of percentile estimates with confidence intervals to determine the central 95^th^ percentile interval (i.e., 2.5th through 97.5th 147 percentile range) for results from > 1000 clinically normal cats ranging in age from 1–3 years collected as part of a wellness study performed by Antech Diagnostics.

In the Long Island, NY laboratory [16] the following results were obtained: For the total T4 assay, the intra-assay coefficient of variation (CV), calculated by assay of 6 replicates of 3 serum pools (with concentrations ≈1.5 μg/dL, 3.0 μg/dL, 10 μg/dL), was 6.5%, whereas the interassay CV, calculated by assay of the same 3 serum pools on 6 consecutive days, was 6.6%. The sensitivity of the (i.e. limit of quantification) of TT4 was 0.5 ug/dl. The reference interval was 0.8–3.8 ug/dl based on 131 normal cats age 7–18 years.

### Statistical analysis

Statistical analysis was performed using JMP 11.1 software program from SAS (Cary, NC). A search was performed to identify data points according to the selection criteria described above. As the data was not normally distributed as determined by the Kolmogorov-Smirnov test, and the health of the patients was not established, the data was evaluated using Tukey’s method [6], which is appropriate for analyzing large univariate data sets. For each data set the median was defined as the middle value of the distribution where 50% of data results are higher and 50% are lower. The interquartile range (IQR) was defined as the range of values that reside in the middle 50% of concentrations between the lower quartile (25^th^ percentile) and upper quartile (75^th^ percentile). The expected distribution of TT4 concentration was defined as being within an upper limit of 1.5*IQR + upper quartile and a lower limit of 1.5*IQR–lower quartile. The Tukey method does not make assumptions about how the population of values is distributed or its mean or standard deviation. “Fences” using multipliers of 1.5 or 3.0 of the interquartile range are termed outliers or extreme outliers respectively. Since it is better for sensitivity of a test to error on being wrong (less specific) than the other way around we used the same thought in choosing the multiple 1.5*IQR- 25% quantile and 1.5*IQR +75% quantile rather than 3.0*IQR.

Values below 0.5 ug/dL were automatically assigned a value of 0.5 ug/dL owing to the lower limit of detection of the TT4 assay. We had 14,463 values reported as < 0.5 ug/dl. This represented ~ 2.5% of the dataset. SAS has found that when dealing with values comprising less than 5% of a dataset, the method of handling limit of detection values does not affect the analysis. When, we evaluated these values by reporting them as either 0.5 ug/dl or as 0.25 ug/dl there was no difference in the results. Outliers were represented by all values outside of the expected TT4 concentration.

The number of outliers in each age group was determined by totaling the quantity of TT4 concentrations above 3.5 ug/dL. The percentage of outliers in each age group was determined by calculating the number of TT4 concentrations greater than 3.5 ug/dL divided by the total number of TT4 concentrations in the respective age group. The rate of change (slope) of percentage of unexpected outliers was determined by calculating the change in percentage outliers divided by the change in age group categories.

## Results

Using the search criteria above, an initial 543,861 TT4 concentrations were identified between January 2014 and July 2015. Data were compiled from a total of 8,673 hospitals in the US. Samples were categorized into the following groups based on sex and reproductive status; female intact (n = 12,672), female spayed (n = 266,532), male intact (n = 10,145), male neutered (n = 254,512). Of the total samples tested, 485,195 samples comprising 89% of the total reference values were categorized as domestic short hair, domestic medium hair, domestic long hair, not identified, unknown, or blank. The remaining 58,666 samples tested comprising 11.0% of the total reference values were represented by 41 different Cat Fanciers’ Association breeds. TT4 concentrations greater than 20 ug/dL (n = 4,135), TT4 concentrations belonging to cats older than 20 years of age (n = 2,309), and TT4 concentrations for which there were no assigned age or obvious misinformation (n = 5,652) were excluded from the data set leaving a total of 531,765 unique TT4 concentrations. In addition, removing cats > 20 years and those with results > 20 ug/dl did not change any of the data conclusions regarding upper fences or outliers.

The data was broken down into age categories including <1 year and years 1 through 20. For each age group, the median value, lower and upper quartiles, IQR, expected TT4 concentration, and outlier values were determined (Table 1).

**Table 1:** Expected and unexpected TT4 concentrations in cats grouped by age. TT4 concentrations in each age group are reported. The values are described by 25^th^ percentile, 50^th^ percentile (median), 75^th^ percentile, IQR, expected TT4 concentration (low and high), and outlier values. The gray shading represents identical values for expected and unexpected TT4 concentrations in cats aged 1 through 9 years. The number of TT4 samples that exceed 3.5 ug/dL, and the percentage of these unexpected outliers in each age group are reported.

						Expected TT4				
						Concentrations				
Age (years)	# Samples	25%	Median	75%	IQR	Low	High	Outlier	# Outliers	% Outliers
<1	2919	1.4	1.8	2.4	1	0.5	3.9	>3.9	193	6.6
1	3,802	1.5	1.9	2.3	0.8	0.5	3.5	>3.5	161	4.20%
2	6,399	1.5	1.9	2.3	0.8	0.5	3.5	>3.5	132	2.10%
3	7,113	1.5	1.9	2.3	0.8	0.5	3.5	>3.5	132	1.90%
4	8,970	1.5	1.9	2.3	0.8	0.5	3.5	>3.5	168	1.90%
5	12,788	1.5	1.9	2.3	0.8	0.5	3.5	>3.5	302	2.40%
6	15,773	1.5	1.9	2.3	0.8	0.5	3.5	>3.5	502	3.20%
7	22,816	1.5	1.9	2.3	0.8	0.5	3.5	>3.5	851	5.70%
8	29,737	1.5	1.9	2.3	0.8	0.5	3.5	>3.5	1,560	5.20%
9	33,896	1.5	1.9	2.3	0.8	0.5	3.5	>3.5	2,245	6.60%
10	46,519	1.5	1.9	2.4	0.9	0.5	3.8	>3.8	4,445	9.60%
11	44,303	1.6	2	2.5	0.9	0.5	3.9	>3.9	5,258	11.90%
12	52,226	1.5	2	2.6	1.1	0.5	4.3	>4.3	7,942	15.20%
13	55,121	1.5	2	2.7	1.2	0.5	4.5	>4.5	9,662	17.50%
14	53,887	1.5	2	2.8	1.3	0.5	4.8	>4.8	10,351	19.20%
15	47,844	1.5	2	3	1.5	0.5	5.3	>5.3	9,860	20.60%
16	36,372	1.5	2	3	1.5	0.5	5.3	>5.3	7,722	21.20%
17	25,005	1.5	2.1	3.2	1.7	0.5	5.8	>5.8	5,676	22.70%
18	15,273	1.5	2	3.2	1.7	0.5	5.8	>5.8	3,946	22.90%
19	7,299	1.4	2	3.3	1.9	0.5	6.2	>6.2	1,748	23.90%
20	3,703	1.4	2	3.4	2	0.5	6.4	>6.4	883	23.00%

For all cats in the data set, the median TT4 concentration was 1.95 ug/dL and the expected distribution of TT4 concentration was 0.5–4.3 ug/dL. For all cats between the age of 1 and 9 years (n = 141,294) the median TT4 concentration, lower and upper quartiles, IQR, expected TT4 concentration, and outlier values were identical (Table 1).

**Fig 1:**
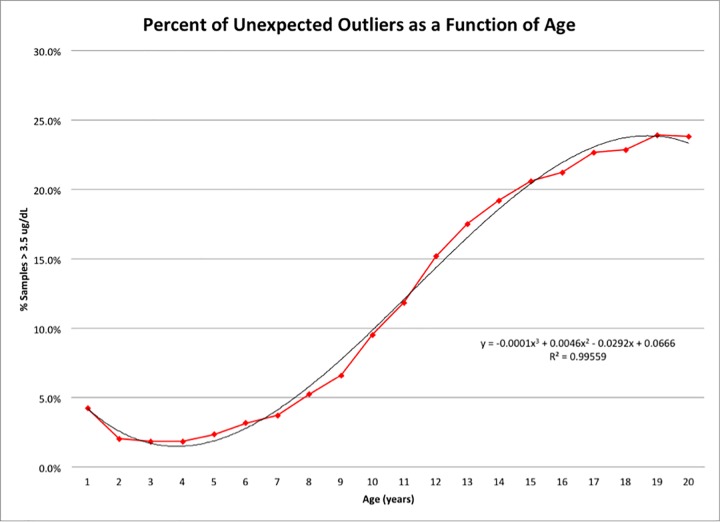
Percentage of unexpected outliers as a function of age. The graph depicts the percentage of unexpected outliers as a function of age (red squares), which is best depicted as a 3^rd^ order polynomial equation (solid black). The 3^rd^ order polynomial equation and R^2^ values are illustrated.

These values were determined to be; median 1.9 ug/dL, lower quartile 1.5 ug/dL, upper quartile 2.3 ug/dL, IQR 0.8, expected TT4 concentration 0.5–3.5 ug/dL, and outlier > 3.5 ug/dL. In cats <1 year of age, the values were determined to be; median 1.8 ug/dL, lower quartile 1.4 ug/dL, upper quartile 2.4 ug/dL, IQR 1.0, expected TT4 concentration 0.5–3.9 ug/dL, and outlier > 3.9 ug/dL. As age increased above 9 years, the distribution of values shifted to the right as these populations contained a greater percentage of cats with higher TT4 concentrations. The number of cats in each age group that had a TT4 concentration greater than 3.5 ug/dL, defining a statistical outlier, as well as the percentage of outliers calculated as the number of outliers divided by the total number of cats of a particular age group were determined (Table 1). The number and percentage of outliers increased with age, particularly in cats over the age of 9 years. A graphical representation of the percentage of outliers as a function of age is shown in Fig 1. The shape of the line representing this relationship was determined to have a 3^rd^ degree polynomial distribution y = -0.0001x^3^ + 0.0046x^2^–0.0292x + 0.0666; R^2^ = 0.9956. Thus, 99.56% of the variation of values outside of 3.5 ug/dL can be explained as a function of age.

**Fig 2:**
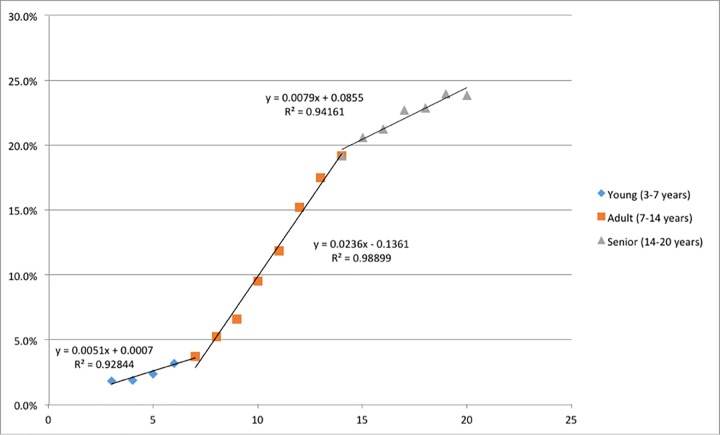
Rate of change of unexpected feline TT4 concentration by age. The graph depicts the slopes for 3 different age groups; young 3–7 years (blue diamonds), adult 7–14 years (orange squares), and senior 14–20 years (grey triangles). Equations calculating the slope of the line and R^2^ values are illustrated.

There were 3 age groups for which the rate of change (slope) of unexpected outliers were identifiable, including 3–7 years, 7–14 years, and 14–20 years. The rate increase was highest from 7–14 years where the slope was 4.6 times faster than from 3–7 years of age. Cats 14–20 years of age had a rate increase that was 1.5 times faster than young cats but 3 times slower than cats aged 7–14 years (Fig 2).

## Discussion

This study differs from previous studies by examining a large population of cats of all ages throughout the United States. As the data was not normally distributed and the health of the patients was not established, the data was evaluated using Tukey’s method which is appropriate for analyzing large univariate data sets and such approaches are distribution independent. By defining exclusion bounds (outliers) rather than the inclusion bounds determined by RI’s, we are able to identify those patients at risk of hyperthyroidism even when their TT4 values fall within the laboratory’s RI.

By examining a total of 531,765 unique TT4 concentrations that were measured using the same validated immunoassay it was shown that for all cats in the data set, the median TT4 concentration was 1.95 ug/dL and the expected distribution of TT4 concentration was 0.5–4.3 ug/dL. This is very similar to the laboratory’s RI of 0.8–4.0 ug/dl which was determined in >1,000 healthy cats between the ages of 1–3 years^15^ and a similar RI of 0.8–3.8 ug/dl found when evaluating 131 healthy, euthyroid cats, over the age of 7 years [16]. This highlights that the use of such large datasets, which may allow for the inclusion of some ill and/or hyperthyroid cats even with the exclusion criteria mentioned previously, in conjunction with appropriate statistical methods, carries sufficient statistical power to demonstrate the homogeneity of the TT4 data.

Perhaps the most notable finding in this paper was that cats between 1–9 years of age had an identical expected distribution of TT4 concentration (0.5–3.5 ug/dL). Cats with TT4 concentrations greater than 3.5 ug/dL represented unexpected outliers in the population even though TT4 concentrations between 3.5–4.0 ug/dl are considered to be within the established RI for the laboratory. Based on this information, the finding of a TT4 between 3.5–4.0 ug/dl, along with historical and physical examination findings suggestive of hyperthyroidism, indicates the need for additional testing or monitoring.

A previous study by Peterson et al [17] which compared TT4 concentrations in cats with untreated hyperthyroidism, non-thyroidal disease, and clinically normal cats demonstrated that there existed a population of cats with mild hyperthyroidism with TT4 concentrations within the RI for the laboratory. These cats typically had a TT4 value greater than 2.3 ug/dl, which was within the laboratory's established RI. Multiple authors have made the recommendation that practitioners consider the possibility of hyperthyroidism when TT4 values are in the middle to upper range of the RI [12–14], however this finding has not been validated in a larger data set, nor has a different statistical methodology been performed to further substantiate that approach. Interestingly, our study which evaluated expected and unexpected TT4 concentrations using a population of 531,765 cats with unknown health status resulted in a similar finding despite the utilization of a completely different statistical methodology and TT4 assay. Based on the results of this very large data set, TT4 values >3.5 ug/dl represent statistical outliers in the population lending support to the recommendation that TT4 values above this cutoff using this TT4 assay should prompt further evaluation by the practitioner. The evaluation of the whole population within an age bracket in detecting outliers represents both physiologic and pathologic conditions. As the number and percentage of outliers was identical through age 9, the change in outliers with increasing age more likely reflects pathology and is consistent with the known natural history of hyperthyroidism and the observed increase in the rate of change (slope) of unexpected outliers with age observed in this study.

The development of RIs in a given population was a concept first introduced in human medicine and later applied to veterinary medicine [18–21]. Laboratory-generated RIs are commonly used to assist in the interpretation of a given observation when the same laboratory technique is used in a corresponding population of animals of the same species.^19^ In veterinary medicine, RIs are generally based on a relatively small sample size. Although there are conventional methodologies for establishing RIs using a small number (i.e. <120) of reference values [5,22], the current study intended to more accurately describe the expected TT4 concentration in cats using a vast data set with greater than 500,000 reference values of TT4. The intent of the present study was not to establish a RI, but rather to utilize the above statistical methodology to determine which TT4 concentrations are expected and which represent outliers, even when such outliers are within the laboratory’s established RI. The samples utilized to determine expected TT4 concentrations in the present study were obtained from cats of all ages and breeds from every part of the US, but without accompanying information regarding health status or clinical circumstance. Generally, when reference values are obtained from individuals in well-defined populations or with established health status, all reference values tend to be retained. Alternatively, in instances where reference values are selected by convenience, where health status is not established or confirmed, or when field methods introduce high levels of inaccuracy, reference values located at extremities are more likely to be excluded [5].

The expected TT4 values were determined using non-parametric methods of percentile estimates with confidence intervals to determine the central 95th percentile interval (i.e., 2.5^th^ through 97.5^th^ percentile range) and the expected TT4 concentrations in the 531,765 cats was determined using Tukey’s method which can be used for data sets where the health status of the population is unknown such as determination of RIs in wildlife species [5,22–23].

Limitations of the present study include its retrospective and observational design. Certain necessary assumptions were made in the present study with regards to the clinical subjects, recording of information, and accuracy of the analysis so that conclusions could be drawn from the data. The first assumption was that younger cats are generally healthy, euthyroid, and without concomitant severe disease that substantially alters their TT4 concentrations when compared to older cats. This assumption is supported by the fact that hyperthyroidism tends to be a disease of geriatric cats. The average age of hyperthyroid cats is 13 years, and the disease uncommonly occurs in cats younger than 10 years of age [3,24–25]. Undoubtedly, a small subset of young cats with elevated TT4 concentrations and clinical signs of hyperthyroidism were included in the current data set, however these values did not meaningfully influence the expected TT4 concentration as cats 1–9 years of age had an identical median and expected distribution of TT4 concentration (0.5–3.5 ug/dL).

An assumption was made about the accuracy of the reported age, sex, reproductive status, and breed by the submitting hospital. While there are recording errors that do occur, the signalment corresponding to the individual data points was accepted to be accurate in the current study. Moreover, while there may have been errors in TT4 concentrations due to pre-analytical errors (i.e. sample handling, shipment, storage), analytical errors, or post-analytical (transcription) errors [5], the TT4 concentrations were assumed to be correct and reflective of the true TT4 concentration in that patient. Additionally, while it was our intent to include only unique TT4 concentrations into the data set, we cannot exclude the possibility of redundant samples in which a specific patient had TT4 concentration performed at two or more separate hospitals using the same reference laboratory during the study period. While these assumptions were not absolute across all measurements, the robust statistical power of this particular data set precludes these exceptions from strongly influencing the overall results. It is important to reiterate that results of the current study pertain only to TT4 concentrations measured by an homogenous EIA method^c^ [15–16,26] at a major commercial veterinary laboratory^b^, and not to TT4 concentrations measured by other assay types (i.e. RIA, chemiluminescent enzyme immunoassay, enzyme-linked immunosorbent assay) [4,27–29]. Despite, the reasonable correlation among varying assay types [30–31], in order for the present interpretations to be extrapolated to other assays, individual diagnostic laboratories would need to perform similar evaluations using their specific, corresponding TT4 assay.

Diagnosis of feline hyperthyroidism relies on compatible clinical signs (i.e. polyuria, polydipsia, polyphagia, weight-loss, altered behavior), identification of a thyroid nodule, and an elevation in circulating thyroid hormone levels. Treatment of hyperthyroidism regardless of modality (i.e. radioactive iodine, pharmacological management with anti-thyroid medications, surgical thyroidectomy, or an iodine-restricted diet) carries a favorable prognosis [15,32–36]. Thus, it is logical to surmise that earlier identification of at-risk patients should improve the overall clinical outcome. The hallmark for diagnosis of hyperthyroidism remains an elevated circulating TT4 concentration, and clinicians rely heavily upon the TT4 RI provided by diagnostic laboratories to make clinical decisions. The majority of overtly hyperthyroid cats have TT4 levels above the individual laboratory’s RI [17–37], although there are also a subset of cats with clinical signs referable to hyperthyroidism that have TT4 concentrations within the “normal” RI, a disease termed occult hyperthyroidism [38]. Based on our data which defines an outlier TT4 concentration as > 3.5 ug/dl, which is lower than the upper end of the RI generated by the reference laboratory (e.g. 0.8–4.0 ug/dL), it is likely that practitioners will identify emerging and occult hyperthyroid cats earlier than they would otherwise, thereby potentially improving the clinical outcome for their patients.

This study demonstrated that age was a strong predictor for the number and percentage of unexpected outliers in the population, accounting for 99.5% of the variation in outlier TT4 concentrations. The greatest change in the number and percentage of outliers occurred in cats aged 7–14 years, which suggests that cats in this age group are more likely to become outliers, and thus should be more closely scrutinized for abnormal TT4 concentrations and clinical signs of hyperthyroidism compared to cats of other age groups. These results are consistent with the observation that hyperthyroidism occurs most commonly in older cats [3,24].

Since its first description in 1979, the incidence of hyperthyroidism has dramatically increased prompting veterinarians and researchers to hypothesize whether exposure to environmental thyroid-disruptor chemicals or other environmental, genetic or dietary factors are involved in the pathogenesis of hyperthyroidism [1]. Potential exposure to several substances have been implicated including organohalogen compounds such as polychlorinated biphenyls and polybrominated diphenyl ethers [39], fertilizers [40], soy isoflavones [38–42], bisphenol-A primarily released from “pop-top” canned cat food lids [43–44], and consumption of commercial canned food [40,43]. One theory as to why the number and percentage of unexpected outliers becomes accelerated over the age of 9 years is that it may take several years of exposure to such environmental, dietary and genetic factors before they express themselves clinically and hyperthyroidism ensues, although this topic requires further investigation.

Based on the current study, the identification of a TT4 concentration greater than 3.5 ug/dL is an unexpected finding and characterizes an outlier in the general population. Irrespective of clinical signs of hyperthyroidism, the identification of an outlier warrants suspicion for the possibility of hyperthyroidism. If the TT4 concentration is greater than 3.5 ug/dL but less than the upper limit of the reporting laboratory’s RI, and the patient exhibits clinical signs of hyperthyroidism, further evaluation is warranted to rule in or rule out hyperthyroidism such as by measurement of free T4 by equilibrium dialysis (fT4ED) or thyroid stimulating hormone (TSH) where a normal TSH concentration can be used to rule-out hyperthyroidism [16]. The identification of a TT4 concentration greater than 3.5 ug/dL in the absence of clinical signs of hyperthyroidism may represent a cat with early or emerging hyperthyroidism. This finding should prompt a high index of suspicion for hyperthyroidism going forward, which may include measurement of fT4ED and/or TSH or close monitoring of that patient’s TT4 and clinical signs in the subsequent weeks to months.

With the establishment of this new expected TT4 concentration in the cat, future studies will focus on determining how breed and geographical distribution within the US affects the expected TT4 concentration. It would be of interest to determine the true incidence and prevalence of hyperthyroidism in the US and different parts of the world, and to evaluate changes in prevalence over time by repeating this study several years from now to determine if the expected TT4 concentration or outlier population changes.
